# Recent Advances in Nanomaterials-Based Targeted Drug Delivery for Preclinical Cancer Diagnosis and Therapeutics

**DOI:** 10.3390/bioengineering10070760

**Published:** 2023-06-25

**Authors:** Harshita Tiwari, Nilesh Rai, Swati Singh, Priyamvada Gupta, Ashish Verma, Akhilesh Kumar Singh, Kajal, Prafull Salvi, Santosh Kumar Singh, Vibhav Gautam

**Affiliations:** 1 Centre of Experimental Medicine and Surgery, Institute of Medical Sciences, Banaras Hindu University, Varanasi 221005, India; harshitaruchi4218@gmail.com (H.T.); nilesh.rai17@bhu.ac.in (N.R.); swati.7672@bhu.ac.in (S.S.); priyamvada.24@bhu.ac.in (P.G.); ashish.4@bhu.ac.in (A.V.); santosh.singh01@bhu.ac.in (S.K.S.); 2 Department of Botany, Institute of Science, Banaras Hindu University, Varanasi 221005, India; 3 Department of Oral and Maxillofacial Surgery, Faculty of Dental Sciences, Institute of Medical Sciences, Banaras Hindu University, Varanasi 221005, India; aksingh.dent@bhu.ac.in; 4 Department of Agriculture Biotechnology, National Agri-Food Biotechnology Institute, Sahibzada Ajit Singh Nagar 140306, India; mittalkajal003@gmail.com (K.); salvi.prafull@gmail.com (P.S.)

**Keywords:** cancer, biomarker, nanotechnology, nanoformulation, nanoimaging, drug resistance

## Abstract

Nano-oncology is a branch of biomedical research and engineering that focuses on using nanotechnology in cancer diagnosis and treatment. Nanomaterials are extensively employed in the field of oncology because of their minute size and ultra-specificity. A wide range of nanocarriers, such as dendrimers, micelles, PEGylated liposomes, and polymeric nanoparticles are used to facilitate the efficient transport of anti-cancer drugs at the target tumor site. Real-time labeling and monitoring of cancer cells using quantum dots is essential for determining the level of therapy needed for treatment. The drug is targeted to the tumor site either by passive or active means. Passive targeting makes use of the tumor microenvironment and enhanced permeability and retention effect, while active targeting involves the use of ligand-coated nanoparticles. Nanotechnology is being used to diagnose the early stage of cancer by detecting cancer-specific biomarkers using tumor imaging. The implication of nanotechnology in cancer therapy employs photoinduced nanosensitizers, reverse multidrug resistance, and enabling efficient delivery of CRISPR/Cas9 and RNA molecules for therapeutic applications. However, despite recent advancements in nano-oncology, there is a need to delve deeper into the domain of designing and applying nanoparticles for improved cancer diagnostics.

## 1. Introduction

Despite continuous progress in the medical field and technology, cancer is still one of the leading causes of mortality worldwide because of its dynamic nature. Cancer generally starts with the formation of a confined benign tumor, which spreads to different parts of the body at later stages by mechanisms such as resistance to apoptosis, contact inhibition, angiogenesis, tissue invasion, and metastasis. In order to metastasize, cancer cells leave their confined primary site, evade the immune cells and attain multidrug resistance to circulate freely in the blood stream [1]. However, cancer is often diagnosed at later stages due to lack of clinical presentation in the early stages, as a result of which the oncogenic cells begin to metastasize in different areas of the body. The frequently used cancer therapies include chemotherapy, radiation, and surgery, which are facing major challenges due to their severe side effects. Chemotherapy may lead to systemic toxicity by showing its action on non-cancerous cells along with the cancerous ones. The side effects of chemotherapy are usually concerned with the central and peripheral nervous systems. Most of the chemotherapeutic regimens often lead to dose-limited toxicity or inactivation of the drug. In general, if chemotherapy side effects are not detected early, they cannot be reversed, which can lead to more severe complications [2]. The potential of chemotherapy can be improved by the application of combinational strategies in chemotherapy. Combinations of more than one drug reduced the uptake of each drug which helps to overcome multidrug resistance. The use of combinational strategies also resulted in synergistic effects and reduced toxicity [3]. Although the development of targeted therapy has advanced existing cancer treatments, ineffective targeted drug delivery has always been an issue because of the failure of the drug delivery at the desired target site, which causes multidrug resistance. The progress of the resistance mechanism in cancer cells and its propensity to relapse are major hurdles in cancer treatment. Thus, more advanced technologies need to be developed to delve deeper into the field of oncology and overcome the havoc caused by cancer.

Nano-oncology is emerging as a cutting-edge field of multidisciplinary research that is expected to outperform traditional cancer monitoring and treatment methods [4,5]. The use of peptides, polymeric materials, ligands, metals, and semiconductors as individual molecules is often not possible in cancer detection and therapy due to their bulky size. However, covalently linking these biomolecules to functional nanoparticles has made a significant contribution to medical applications, molecular profiling, and biological assays. Nanotechnology is a boon in the current era; it can be used in early diagnosis and management of different types of cancers and oral squamous cell carcinoma is one of them. Oral squamous cell carcinoma (OSCC) is a chronic debilitating disease and the most common malignancy of head and neck region. Several nanoformulations have been designed to overcome the common side effects of conventional drugs like bone marrow depression, nephrotoxicity, ototoxicity and poor systemic stability. A ligand decorated cancer targeted CDDP-loaded poly lactico-glycolic acid-polyethylene glycol (PLGA-PEG)/NR7 nanoparticle was reported to exhibit fast intracellular infiltration and has better capacity of drug penetration in OSCCs tissue [6]. Similarly, Ferumoxtran-10 is a lymphotrophic NP used as MRI contrast agent. It is a biodegradable ultra-small superparamagnetic iron oxide (USPIO) covered with low molecular weight dextran with a diameter of 17–21 nm [7]. It has a long blood half-life and can easily enter the iron metabolism through transferrin, ferritin, hemosiderin, and hemoglobin. It can easily cross the capillary wall, enter the lymphatic fluid and become accumulated in the lymph nodes. Hafnium oxide NPs (NBTXR3) has been investigated in association with IMRT (Intensity modulated radiotherapy) in elderly patients of OSCCs. It has the property of increased cell localized energy deposit upon RT exposure [8]. Recently, silver-based nanoparticles were investigated in oral cancer, colon cancer, breast cancer, liver cancer, lung cancer and in glioblastoma; they have the property to inhibit the proliferation of cancer cells by direct DNA damage and cell cycle arrest in S-phase leading to cell death [9].

**Figure 1:**
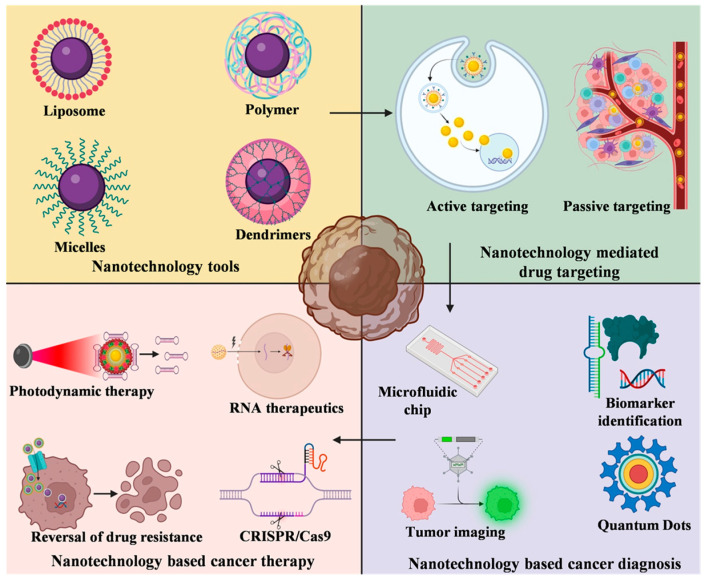
Nanotechnology tools-based cancer targeting diagnosis and treatment. Various types of nanotechnological tools such as liposome, polymers, micelles, and dendrimers are exploited for targeted delivery of drug nanoparticles. The targeting of these nanoparticles may be active or passive. Nanotechnology-based cancer diagnosis is possible by the application of microfluidic chip, identification of biomarkers, nanoscale probe and quantum dots. Cancer treatment is made possible by nanotechnology-based photoinduced gene silencing, RNA mediated cancer nanotherapy, overcoming multidrug resistance and nanotechnology-based CRISPR/Cas9 delivery.

Apart from this, lymph node and prostate cancer detection and targeted gene delivery are made easier by the use of paramagnetic iron oxide nanoparticles and polymeric nanoparticles, respectively [10,11]. Zinc sulfide-coated cadmium selenide nanoparticles are used during tumor surgery due to their property of glowing in the presence of ultraviolet light after seeping into the tumors [12]. Metallic and magnetic nanoparticles prepared from silver, gold and iron oxide, respectively, are conjugated to peptide molecules. The peptide-metallic nanoparticle conjugates are reported to be biocompatible, least cytotoxic and play a substantial role in *in vitro* cancer diagnosis. Nanoparticles are administered intravenously in the blood where they interact with the multiplex surrounding that has evolved to eradicate the foreign materials. Phagocytic cells recognize the nanoparticles as foreign agents and uptake them as soon as they enter blood stream and undergo opsonization. The adequate quantity of drugs and nanoparticles could not be delivered uniformly to the tumor because of barriers such as antigen expression and heterogeneous surrounding of the tumor and its permeability. Hence, the field of nanotechnology needs an up-gradation in such a way that it can escape body clearance and show specific tumor accumulation. The significance of nanotechnological tool and their advances in drug delivery and modes of targeting cancer cells including the reversal of multidrug resistance illustrated in Figure 1. Furthermore, this article aims to shed light on the use of nanotechnology for detecting precise cancer biomarkers and tumor imaging. In addition to this, the use of nanotherapy in cancer treatment and upcoming challenges and perspectives related to nano-oncology are also discussed further.

## 2. Nanomaterials-Based Approaches in Oncology

Diverse ranges of tools are offered by nanotechnology, including nanochips, nanoscale probes, liposomes, polymeric micelles, carbon nanotubes, nanocantilevers, quantum dots, and dendrimers (Table 1). Sensor test chips contain multiple nanowires that have the potential to modify individual cells and detect the biomarkers of oncogenic cells, which help to diagnose cancer at its initial stages even from a small amount of blood sample. Nanoscale probes are used to track complex molecular and cellular events in real time, which helps to monitor and manipulate cancer cells and identify the genetic and biomolecular targets for future therapies. In addition to this, the use of nanolabels are expected to show advancements in gene expression studies and high-throughput screening. The property of the nanoparticles to adopt a wide range of shapes and sizes based on their manufacturing process makes them suitable to be used as drug carriers such as liposomes, polymer drug conjugates, and immunoconjugates.

### 2.1. Lipid-Mediated Nanoformulations of Anti-Cancer Drugs

Liposomes have emerged as a versatile tool in the field of nanotechnology because of their unique structure and composition. Liposomes are self-assembling, spherical, and colloidal structures in which a central aqueous space is surrounded by a lipid bilayer. The use of immunoliposomes, in which an antibody is conjugated at the extreme outer surface of PEGylated liposomes, has been remarkable in terms of the internalization of antigen and specific targeting [27]. On the other hand, the treatment of Kaposi’s sarcoma and breast cancer is made possible by the liposomal formulation of anti-cancer drugs such as doxorubicin (DOX) and daunorubicin (DU) [28,29]. It has also been observed that a PEGylated liposomal formulation of DOX (MCC-465) targets metastatic stomach cancer [30], and melanoma cells also show increased intracellular absorption of the liposomal formulation of DOX in comparison to the free DOX that leads to the destruction of cancer cells [31]. A recent study investigated the *in vivo* anti-cancer efficacy of mycophenolic acid (MPA) and quercetin (QC) co-administered using liposomal nanoparticles (LNPs). MPA-QC NPs were reported to exhibit improved breast cancer treatment in SD-rat models [32]. Moreover, the synthesis of a targeted nanoparticle by conjugating LinTT1 peptide with sorafenib (SRF) and DOX co-administered in therapeutic liposomes demonstrated increased cytotoxicity and precise p32 targeting against breast cancer cells [33]. Additionally, liposomal nanoformulation of DOX and ICG was done through their PEGylation and further coated with polydopamine (PDA), which resulted in the formation of PDA@Lipo/DOX/ICG nanoparticle. This nanoparticle was reported to exhibit remarkable anti-cancer therapeutic efficacy after being subjected to irradiation for PDT [34]. Lipid nanoparticles (LNPs) have also emerged as one of the most cutting-edge methods for the effective *in vivo* administration of exogenous mRNA vaccine. Further, by enhancing the CD8 response, 113-O12B encapsulated with TRP-2 peptide (TRP2180-188)-encoding mRNA has also been reported to display excellent tumor suppression activity [35].

### 2.2. Polymer-Mediated Nanoformulations of Anti-Cancer Drugs

Biodegradable polymers are being used frequently in oncology. Nanoparticles are colloidal particles coated with hydrophilic polymers in which the desired therapeutic drug is either encapsulated or covalently conjugated on their surface. The coating is done to bypass the MPS, as the macrophage would consider the uncoated nanoparticles as foreign agents, which would result in their elimination from the bloodstream. The polymeric coating also prevents the nanoparticles from enzymatic degradation and ensures their biocompatibility. Coating polymers can be natural (heparin, chitosan, albumin) or synthetic (poly-l-glutamate, poly-l-lactide, PEG). The advantage of the smaller size of polysorbate-coated nanoparticles overcome the limitation of blood-brain barrier and enables the release of the drug at the target site in the brain after intravenous injection [36]. Currently, several anti-cancer drugs like paclitaxel poliglumex (Xyotax), HPMA, and MAG-CPT have been subjected to polymeric formulations to show increased therapeutic efficacy. The albumin-coated polymeric formulation of paclitaxel (Abraxane) has recently shown better response in the treatment of aggressive breast cancer in comparison to free paclitaxel [37,38]. It was also observed that HPMA-DOX showed alleviation of anthracycline-related toxicity without losing its anti-cancer activity [39]. Recently, it was observed that when rapamycin was loaded into the nanoparticles after conjugating it with an EGF antibody, it showed a better therapeutic response against MCF-7 breast cancer cell lines [40]. The polymeric nanoformulation of curcumin was done using glycerol monooleate and pluronic F-127, which prevents it from hydrolytic degradation and makes it soluble to ensure its delivery at the target site [41]. Similarly, doxorubicin, a potent anti-cancer drug, also showed improved therapeutic efficacy when targeted to the nucleus of breast cancer cells after being subjected to surface modification by the conjugation of a nuclear localization sequence [42,43]. Additionally, photosensitizer-loaded H_2_O_2_-responsive polymer prodrug NPs offer a sustainable method to improve chemo-photodynamic synergistic cancer therapy [44]. Dendrimers and polymeric micelles are the two main modifications of polymeric nanoparticles.

#### 2.2.1. Dendrimers

Dendrimers are hyperbranched macromolecules with radial symmetry and a precise structure consisting of a symmetric core, an inner shell, and an outer shell. These are the self-assembling, polyvalent nanoparticles whose size and structure can be altered, which makes them suitable to be used as drug delivery agents. Due to their polyvalent property, dendrimers are capable of attaching several imaging probes and hence can serve as an excellent tool for the cancer diagnosis. Recently, DNA-assembled polyamidoamine dendritic clusters have shown encouraging results in the specific targeting of cancer cells [45,46]. Furthermore, the photothermal and photodynamic treatment efficacy of photosensitizer-decorated confeito-like gold nanoparticles for cancer therapy has been demonstrated to be enhanced by nanoscaled polyamidoamine dendrimer spacer [47]. On the basis of an amphiphilic penetrating peptide; BL Oc-SS-CPT, a newly synthesized ETCPP (enzyme-triggerable cell penetrating peptide) dendrimer conjugated with a camptothecin (CPT) was developed and it exhibited a remarkable anti-cancer potential [48]. Recently, a manganese-based and hypericin-loaded polyester dendrimer nanoparticle (MHD) was designed to improve cancer detection by MRI and hypericin-based photodynamic therapy (PDT) [49]. The emphasis has shifted to DOX targeting via nanotechnological tools such as dendrimers, in the core of which the drug can be loaded and targeted [50].

#### 2.2.2. Polymeric Micelles

Polymeric micelles are shell structures made up of amphiphilic polymer blocks and are emerging as the latest class of macromolecular nanoscale delivery. Genexol-PM is the first polymeric micelle alteration of paclitaxel and is devoid of cremophor [51]. The polymeric micelle formulation of DOX has also shown promising results in the treatment of restenosis [52]. It has also been observed that the co-administration of the drugs cisplatin and paclitaxel in polymeric micelles has resulted in synergistic effects and enhanced anti-tumor activity in comparison to their individual delivery in free form [53]. The fabrication of DOX-loaded polymeric micelles coupled with nitrogen-doped carbon dots for targeted breast cancer treatment has also been the subject of recent research [54]. Glycol chitosan (GC) and phenylboronic acid were chemically conjugated to create an antiglycolytic amphiphilic polymer that is intended to regulate glucose metabolism while taking into account the distinct metabolism of cancer cells (Warburg effect). In physiological conditions, derivatives of GC-PBA exhibit the capability to create stable micellar structures and demonstrate responsive characteristics when exposed to variations in the concentration of glucose [55]. It has been reported that an amphiphilic polymer T1 may self-assemble and form stable micelles in aqueous solution. This polymer has a tetraphenylethene unit and a poly(ethylene glycol) chain connected by an esterase-responsive phenolic ester bond. DOX, a hydrophobic drug, was successfully encapsulated within the micelles, and in the presence of esterase, it was possible to detect the selective disintegration of drug-loaded T1 micelles. T1@DOX micelles had positive therapeutic effects on HeLa cells, whereas normal cells were mostly unaffected. When compared to DOX-HCl, T1@DOX micelles substantially inhibited the transformation of malignant tumor cells and had less adverse effects, according to *in vivo* anti-cancer studies [56]. Thus, the emergence of several novel approaches to exploit nanoparticles for targeted tumor imaging and drug delivery has made an important contribution to the field of oncology.

### 2.3. Nanotherapy in Drug Delivery

Nanomaterials are being exploited as drug delivery systems because of their large surface area, surface and interface effects, and good specificity. Surface modification of nanoparticles can prevent them from macrophage attack, thus ensuring a better target. Polyvinylpyrrolidone and PEGylated nanoparticles are widely used to target specific tumor sites with anti-cancer drugs [57]. For the first time, gelatin (G)-polyvinylpyrrolidone (PVP) coated graphene oxide (GO) nanocarriers were synthesized. A double nanoemulsion water/oil/water with bitter almond oil was designed as a barrier surrounding the nanocomposite to regulate controlled drug delivery for cancer therapy after the QC drug was loaded into the nanocarriers [58]. *In vitro* and *in vivo* studies have also revealed that a potential long-acting polysaccharide-based platform for anti-cancer research is provided by DPCS encapsulated with DOX and IND. In the study, hydrogels made of chitosan and dextran phosphate were designed to deliver DOX and indomethacin to target sites [59]. The synergistic effect of both of the drugs was reported to induce enhanced apoptosis in tumor cells by alleviating the level of MDR-associated proteins. Nanomaterials such as Fe_3_O_4_ NPs, ZnO NPs, and carbon nanotubes are used to deliver some common chemotherapeutic drugs such as DU and DOX. Numerous studies have been carried out to decipher the interface of nanotechnology and stem cells for gene therapy, cell-based therapy, and regenerative medicine [60]. In the instance of acute lymphocytic leukemia, leukemia cells displayed enhanced vulnerability to doxorubicin-induced cell death. The drug’s ability to suppress serine/threonine WEE1 kinase expression utilizing chitosan-carboxymethyl dextran-polyethylene glycol-TAT nanoparticles provided evidence that it may be used as a potent anti-cancer therapeutic [61]. Isolation of cells from the tumor microenvironment is crucial in stem cell-based cancer therapy. A growing body of literature suggests the advances in the isolation of a single cell and identification of cancer cell-specific biomarkers such as laser capture microdissection, micromanipulator, magnetic-activated cell sorting, microfluids and fluorescence activated cell sorting (FACS) [62]. Magnetic labelling-based isolation of a cell is a widely used method to harvest cancer stem cells from a cell suspension [63]. In addition to this, a study showed the designing of nanoprobes using different components such as magnetic cores, fluorescent agents, targeting antibodies and surface-enhanced Raman scattering generators to facilitate precise and rapid isolation of cancer cells, which substantially impacted the diagnosis of cancer [64]. Overall, the use of lipid-mediated and polymer-mediated nanoformulations of anti-cancer drugs, along with nanotherapy in drug delivery, has significantly improved targeted drug delivery, enhanced therapeutic efficacy, and reduced side effects on healthy cells. Moreover, nanoparticle-based formulation of drugs offers great promise for improving cancer treatment outcomes.

## 3. Mechanistic Insights for Targeted Drug Delivery

**Figure 2:**
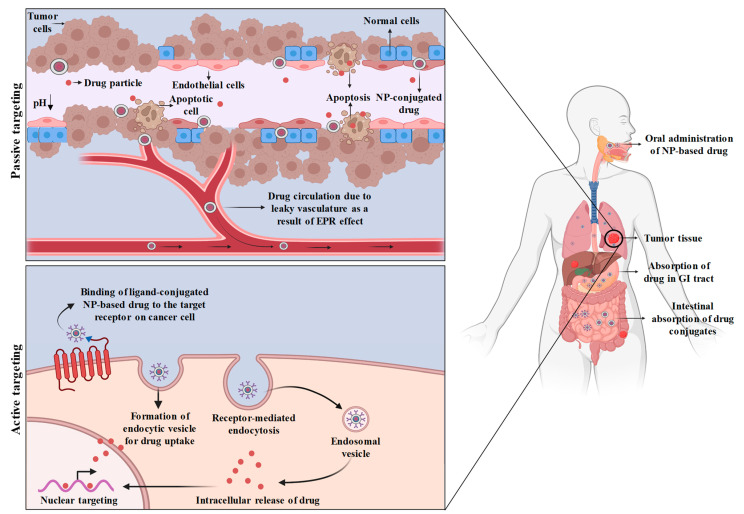
Mechanism of passive and active targeting of NP-drug conjugates. During passive targeting, the drug-loaded NPs are transported via leaky blood vasculature through EPR effect due to poor lymphatic drainage and release of drug from the nanoparticles takes place due to acidic microenvironment of tumor. On the other hand, active targeting involves binding of ligand-conjugated drug-loaded nanoparticles to the antigenic receptor on cancer cells followed by receptor-mediated endocytic uptake.

Efficient targeting of cancer cells through nanoparticle-based drug delivery is important for enhanced therapeutic efficacy, reduced dose-limited toxicity, and protection of normal cells from systemic toxicity. Anti-cancer agents should be targeted to the tumor site using nanoparticles in such a way that they reach the tumor tissues while passing through all of the phagocytic barriers with minimal loss of blood activity or volume. Furthermore, the active form of the drug, after being administered intracellularly, should only act on the cancerous cells without affecting normal cells. The interaction between nanomaterials and tumor cells is currently an intensive research topic that aims to delve deeper into the field of nanoparticle-based targeted drug delivery. Mechanisms of drug delivery can be grouped into two types, the first one is driven by non-ligand mediated nanoparticles i.e., passive targeting, while the later one, active targeting is mediated by ligand conjugated nanoformulations (Figure 2).

### 3.1. Passive Targeting

The delivery of the drug through a drug carrier system by passive targeting at the desired site depends on physico-chemical or pharmacological factors. Passive targeting considers intrinsic nanoparticle properties such as size and chemistry, as well as tumor vasculature, the enhanced permeability and retention (EPR) effect, and the microenvironment of tumor. The stimulation of angiogenesis takes place when tumor tissues require nutrients and oxygen. Tumor cells send signals by releasing molecules so that new blood vessel formation starts in the surrounding host tissues. For instance, FENDRR; a long non-coding RNA (LncRNA) promotes the proliferation and angiogenesis of human retinal endothelial cells in the presence of high glucose [65]. The gaps present between the angiogenic blood vessels are wider than the ones present between the normal cells. Due to faulty vasculature, inadequate lymph is drained, which leads to the induction of the EPR effect. This vascular embolism permits the extravasation of the nanoparticles through extravascular spaces, where they accumulate inside tumor tissues. The size of the nanoparticles should not be more than 100 nm, and the presence of hydrophilic surface helps to cope with the MPS clearance [66]. Passive targeting could also be achieved by covalently linking PEG with the nanoparticles, which have the potential to circumvent ligand desorption and aggregation when released in the bloodstream [67]. It has been discovered that increased concentrations of vascular mediators such as bradykinins, prostaglandins, nitric oxide, basic fibroblast growth factors, platelet-derived growth factor, VEGF-A, and TGF-βfamily members are responsible for leaky and defective vasculature during angiogenesis [68]. Nanoparticle-based drug delivery has enhanced efficacy and drug accumulation over free drug delivery. The factors that influence the distribution of nanoparticles inside the tumor tissue include circulation, half-life, surface characteristics, size, and the level of angiogenesis.

The microenvironment surrounding the tumor cell is yet another factor that contributes to passive targeting. Tumor cells need a high metabolic rate for their rapid growth and hyperproliferation. However, the nutrient and oxygen supply of the body is usually not enough to meet the requirements of tumor tissue. Thus, tumor cells use hypoxic metabolism (glycolysis) as a source of extra energy, which results in a lowering of the pH and creates an acidic environment [69]. Liposomes as a source of drug delivery nanoparticles are designed in such a way that they remain at a pH of 7.4 but become degraded in the acidic surrounding of tumor cells, resulting in the liberation of the active drug [70]. Matrix metalloproteinases (MMPs), one of the enzymes produced by cancer cells, are responsible for the growth, development, and migration of these cells and hence can influence angiogenesis and metastasis [71]. Therefore, it has been observed that targeting MMPs in cancer cells through tumor-activated prodrug therapy has enhanced cancer treatment efficacy [72]. A potent anti-cancer drug, doxorubicin, when bound to albumin, incorporates a MMP-2-specific octapeptide sequence (Gly-Pro-Leu-Gly-Ile-Ala-Gly-Gln) that was observed to be cleaved by the enzyme, which resulted in its conjugation with the DOX tetrapeptide (Ile-Ala-Gly-Gln-DOX), and ultimately DOX is released [73]. However, heterogeneous localization of the nanoparticles inside the tumor cell is still a matter of concern and one of the major drawbacks of passive targeting. Other than drug outflow from the gaps between the angiogenic blood vessels, the factors that impede homogeneity and are responsible for the irregular distribution of nanoparticles inside the tumor are yet to be known.

On the other hand, dynamic covalent bonds have emerged as a powerful mode of passive drug targeting for cancer therapy. Drug molecules chemically bonded to polymer side chains by either a weak bond or a dynamic covalent connection are known as polymer-drug conjugates. When these conjugates enter the blood circulation system, they take on the inactive form of prodrugs. A prodrug is a type of medication that is inert while being transported to the site of action and becomes active once it reaches the desired location. Prodrug reconversion, or turning a prodrug into its active form, takes place inside a particular organ, tissue, or cell. Prodrug reconversion is often accomplished by regular metabolic processes, such as the breakdown of a drug-polymer link by certain cellular enzymes. Recently, the delivery of paclitaxel drug by its covalent conjugation with PEG-dihydrolipoic acid through a disulfide linkage (MeO-PEG2k-DHLA) has also shown anti-cancer activity against 4T1, A549, BEL-7402, and MCF-7 cancer cell lines [74]. Moreover, the concept of reversible pH-regulated drug release was proposed, which emphasized the delivery of active cargo-DOX based on dynamic covalent interactions inside lipophilic drug sponge nanocomposites [75]. Thus, the selective release of the medication to the targeted tissue and the reduction in chemotherapy’s frequent adverse effects have been made possible by passive drug targeting through dynamic covalent bonding, thereby enabling a more specific application [76].

### 3.2. Active Targeting

The mechanism of active targeting is much more advanced than that of passive targeting. Active targeting makes the advantage of an antibody or ligand-conjugated nanoparticles that specifically binds to the antigen that is expressed by tumor cells. The active targeting approach, which makes use of ligand-linked nanoparticles, emphasizes delivering the drug with better efficiency and more specificity. Active targeting makes use of interactions between the targeting moieties and the corresponding receptor. These targeting moieties regulate the attachment of the conjugated ligand to the differentially expressed receptors present on the surface of the tumor cell. It has also been revealed that the presence of multiple binding sites on the targeting moiety ensures better biorecognition and receptor-mediated endocytosis. The use of antibodies as targeting moieties is generally preferred due to recent progress in genetic engineering and molecular biology. Monoclonal antibodies are the most frequently used ligands for active targeting approaches. The interactions responsible for active targeting are broadly categorized into three main classes: carbohydrates-lectin, antibody-antigen, and ligand-receptor, whereas the targeting moieties can be carbohydrates, antibodies, peptides, or small molecules.

#### 3.2.1. Carbohydrate-Conjugated Drug Nanoparticles

Because of their specific and reversible binding with endogenous lectins present on the surface of tumor cells, carbohydrate-linked polymer-drug conjugates have been used for targeted delivery in recent years. Carbohydrates mainly include glycoproteins, glycolipids, and polysaccharides, while lectins include diverse classes of proteins that are specific for carbohydrates. Carbohydrate-conjugated nanoparticles, after binding to the lectin-specific tumor cells, undergo lectin-mediated endocytosis to give the desired therapeutic result. Once the drug-polymer conjugate is uptaken intracellularly, glycoproteins cannot eliminate them, due to which this approach of active targeting gets a grip on multidrug resistance. In a recent study, Dox and fucoidan were covalently conjugated to design a new P-selectin-specific drug delivery system for the treatment of breast cancer [77]. Studies have also unveiled the development of anti-viral nanocarriers for siRNA mediated cancer treatment by the application of synthetic methods coupled with cyclodextrin (CD) conjugated carbohydrate polymers [78].

#### 3.2.2. Antibody-Conjugated Drug Nanoparticles

With recent progress in antibody research, a diverse range of antibodies with high specificity and exceptional binding affinities are being used as a means of active drug delivery for targeted cancer therapy. Monoclonal antibodies can efficiently transport drug nanoparticles to target tumor sites where they can attach specifically on the antigen receptors expressed by lung cancer cells [79]. The use of whole antibodies is generally less preferred because the F_C_ domain of mAbs can generate an immune response, which can result in their fast elimination from the blood circulation system. Conversely, using an antibody fragment as a targeting component can decline immunogenicity and clearance rate, increasing circulation half-life. *In vitro* and *in vivo* studies have also emphasized a simultaneous heteroreceptor cross-linking of CD20 and CD38 receptors by non-toxic macromolecular therapies to destroy B-cells for treatment of leukemia [80]. The conjugation of antibodies to nanoparticles brings the drug into close proximity with the tumor cells to induce enhanced anti-cancer activity. For example, the conjugation of HPMA-copolymer mesochlorin e6 (Mce6) to the carbohydrate domain of the OV-TL16 antibody resulted in better affinity and improved homogeneity as compared to its conjugation with the amino group of the antibody [81]. Therefore, it is important to examine the effect of various conjugating techniques to enhance the potential anti-cancer activity.

#### 3.2.3. Peptide-Conjugated Drug Nanoparticles

Peptides are linked to the drug in the form of a short peptide sequence, in which a chain of three to fifteen amino acids are joined together by a peptide bond. The oligopeptide sequence is highly specific for its receptor, which is uniquely expressed by the tumor cells. The polypeptide known as RGD, composed of arginine, glycine, and aspartic acid was the first cell-targeting polypeptide identified, with a strong affinity for α**_v_**β**_3_** integrins, that are overexpressed by both tumor cells and angiogenic endothelial cells [82]. It has been found that RGD-mediated drug targeting has enhanced the efficacy of targeted drug delivery, resulting in a better therapeutic approach. However, cyclization and multimerization of the RGD have resulted in better interaction of the peptide with the target receptor [83].

Studies have also demonstrated that small molecules such as PD-1 (programmed cell death protein)/PD-L1 (programmed death ligand-1) conjugating peptides have drawn a lot of interest in cancer immunotherapy because of their potential to disrupt PD-1/PD-L1 interactions [84]. As an anti-PD-1 peptide with a twenty-nine amino acid sequence, NP-12, for instance, was found to collaborate closely with ICD (immunogenic cell death) to provide successful ICB (immune checkpoint blockade) immunotherapy. When paired with chemotherapeutic agents that cause ICD, the anti-peptide of PD-L1 (CLQKTPKQC) efficiently prevented the PD-1/PD-L1 interactions by having a high binding affinity for PD-L1 and revitalized the T-cells, leading to strong ICB immunotherapy. Yet, because of their significant proteolytic cleavage and brief *in vivo* half-lives, such PD-1/PD-L1 interacting peptides have demonstrated negative therapeutic effectiveness. Anti-PD-1 or anti-PD-L1 peptides have been widely used in multifunctional nanomedicines such as polymeric nanoparticles, liposomes, and dendrimers to circumvent these disadvantages.

In a recent study, trans-activator of transcription (TAT) peptide, a cell-penetrating peptide, was conjugated to silver nanoparticles made from the cell-free extract of *Staphylococcus aureus* in order to improve the induction of apoptosis in breast cancer cells [85]. Furthermore, C_7_H_2_ and HuaL peptides were conjugated synergistically to AuNPs to enable dual-peptide targeting of human foreskin fibroblast (Hs68) cell line and malignant melanoma cell line *in vitro* (B16F10-Nex2) [86]. Considering the advantages of peptides such as minute size, lower immunogenicity, higher stability, better specificity, and strong binding affinity with the drug nanoparticles, peptide-conjugated nanomaterials could provide better therapeutics against cancer and other diseases.

## 4. Cancer Diagnosis Using Nanotechnology

Cancer diagnosis is heavily based on tissue morphology at the microscopic level from a biopsy or surgically obtained tumor. Histopathology analysis is done using hematoxylin, and the histomorphology of the eosin-stained tissues is evaluated by electron microscopy [87]. Additionally, bioconjugated particles are being developed for the prior cancer detection of body fluids, including serum and blood [88]. Cancer-specific antibodies or other ligands are frequently coated on the sensors to provide an electromechanical or optical signal that may be used for detection when a cancer cell or target protein is captured [89]. The application of nanoparticles for the detection and evaluation of circulating cancer cells or their biomarkers present in blood and serum samples is another encouraging area of research, as they are often present in very low concentrations [90,91]. However, it is conceivable to improve the capability of capturing and analyzing these uncommonly circulating cancer cells by combinatorial use of magnetic nanoparticles and semiconductor quantum dots [92]. Thus, for cancer treatment and surveillance, nanotechnology may benefit from the distinct architecture, vascularity, antigenicity, biomarkers, and microenvironment of tumors.

### 4.1. Identification of Biomarkers for Detection of Cancer

An oncogenic biomarker is a biomolecule that may be found in the body’s tissues and fluids and can be used to recognize the presence of cancer cells. Cancer cells secrete proteins, carbohydrates [93], or nucleic acids as biomarkers during carcinogenesis [94]. The evaluation of the biomarker level aids in the early detection of cancer and its possibility of relapse, which in turn improves related monitoring and therapeutic efficacy. Hereby, the subsequent sections emphasize the selected oncogenic biomarkers used in nanotechnology-based cancer diagnosis.

#### 4.1.1. Protein Biomarkers

The recognition of the protein biomarker is facilitated by selective interactions with antibodies, their fragments, or aptamers [95]. After successful identification, the interaction event is subsequently converted into a quantifiable signal. In recent studies, cancer biomarkers have identified using QD-based biosensors. Wide absorption, anti-photobleaching property, great degradation resistance, high molar extinction coefficient and quantum yield are some of the distinctive qualities of QDs [96]. Protein biomarkers such as CEA [97], AFP [98], PSA [99], and CA-125 [100] have been approved for detecting colorectal, liver, prostate, and ovarian cancers, respectively. QD-conjugated aptamers [101] and QD-embedded silica nanoparticles are used for the detection of the CA-125 biomarker for ovarian cancer and the PSA antigen for prostate cancer, respectively, using the lateral flow technique [102].

#### 4.1.2. MicroRNA (miRNA) Biomarkers

The single-stranded RNA (ssRNA) with a length of 20–22 nucleotides, known as micro-RNAs (miRNAs), have the ability to attach to an mRNA and inhibit its translation. A single mRNA can be targeted by multiple miRNAs, and a single miRNA can also influence multiple gene expressions. The gene expression that regulates cell growth, division, and apoptosis is regulated by these genetically encoded molecules. As the production of miRNAs is downregulated, normal cellular function becomes impaired, ultimately leading to cancer. The regulation of miRNAs is influenced by oncogenic viruses in a variety of ways [103], including upregulating the host miRNA to stimulate the division of virally infected cells, encoding viral miRNAs that target the mRNA of the host cell, and reducing the miRNA of the host that restricts tumor growth. Electrodes for measuring miRNA-21 concentrations in the serum of breast cancer patients are modified using AuNPs@NIPAm-co-AAc microgel nanocomposites [104]. The SERS platform, which is based on nanotechnology, offers a precise and focused investigation of the miR-29a biomarker for cancer diagnosis [104]. A simple blood sample is adequate to detect the presence of miRNAs because miRNAs are not affected by the RNase enzyme and, hence, circulating miRNAs are more stable. Therefore, for early diagnosis and therapeutic monitoring, many organ-specific or tissue miRNA indicators can be utilized.

#### 4.1.3. Circulating Tumor DNA Biomarkers

The majority of risks from solid tumors are caused by metastasis. In the course of metastasis, a cancer cell from the benign tumor first attacks the tissue around it before undergoing intravasation in blood and lymphatic system, where it survives and spreads to microvessels of other tissues. These cells ultimately leave the circulation (extravasation) and continue to exist in the distant milieu, which creates an ideal foreign microspace for the growth of new tumors. About 100–200 base pair-long tumor-derived DNA fragments flow in the bloodstream as circulating tumor DNA (ctDNA). These ctDNA are released from primary and circulatory tumor cells (CTCs), which provide a valuable means to detect cancer-related genetic abnormalities, enabling the diagnosis of cancer even before symptoms manifestation. CTCs have been thoroughly studied as a component of liquid biopsy [105]. The primary targets are the distinctive surface proteins of CTCs, such as EpCAM [106], which can be used as an oncogenic biomarker because it has been demonstrated in numerous studies that EpCAM is overexpressed on CTCs from different types of human cancers, leading to the frequent use of anti-EpCAM compounds in CTC screening. Recently, the detection of ctDNA of lung cancer was made possible by the application of NG-PEI-COFTAPB-TFPB as a sensing tool and Fe-MOF for signal amplification [107]. Nanotechnology has also been exploited for the advancement of certain immunoassays such as DNA biosensors and microfluidic chips, which enhance their selectivity and specificity.

### 4.2. Microfluidic Chip

A microfluidic chip is another high-throughput analytical technique that is used because it has good detection efficiency, consumes less energy, has a shorter detection time, and is cost-effective. The detection efficiency of the microfluidic chip is thirty times higher than that of ELISA. A microfluidic chip is also used for the detection of tumors at their early stages, after which treatment can be done at the initial level so as to avoid further complexities. Studies have demonstrated that a nanotechnology-based microfluidic immunoarray device, CoZnFeONPs offers a straightforward and efficient approach for biomarker identification that might meet the demand for a quick, low-cost test for early cancer diagnosis [108]. The immunoassay microfluidic chip is also used for the identification of CEA antigen in human blood [109] Recently, the application of a microfluidic chip-based MRS immunosensor was also validated for cancer biomarker detection via enzyme-mediated nanoparticle assembly [110].

### 4.3. Quantum Dots Mediated Nanoformulations in Cancer Diagnosis

The exploitation of semiconductor quantum dots as nanoprobes is achieving great heights in the field of medical science because of their excellent fluorescent property. The technological significance of quantum dots makes them suitable to be used for cellular imaging. QDs need only a single excitation source to execute multiplexed imaging due to their wide absorption and narrow emission properties. QDs exhibit quantum mechanical characteristics and emit a particular fluorescent color when excited with a light source such as UV light. The extension in wavelength of QDs has resulted in enhanced tissue penetration and minimized background fluorescence. *In vivo* imaging depends on the Stokes shift, which varies according to the wavelength of the excitation light. QDs have higher photostability and an intense fluorescence property, due to which they are outperforming organic fluorescent dyes and proteins. The brightness of QDs in photon-limited conditions is better than that of organic dyes because of their larger molar extinction coefficient. The application of QDs as a classic fluorescent probe and nanocarrier material has contributed largely to early cancer detection and the study of signal transduction and cell localization. According to a recent study, it has been found that surface-modified CdTe QDs boosted the anti-tumor activity of the drug against the oncogenic cell [111]. In addition to this, intravenous injection of PEGylated QDs conjugated with monoclonal antibodies resulted in the specific targeting of tumor tissue [112].

### 4.4. Real-Time Cancer Monitoring Using Nanoimaging

Clinical oncology relies heavily on tumor imaging, with radiological tests able to identify solid tumors, ascertain recurrence, and track therapy outcomes. The primary goal of traditional tumor imaging techniques like CT and MRI is to delineate the morphological characteristics of the tissue, tumor, and organs, including the anatomic site, extent, and tumor size, at different angles of spatial resolution and contrast [113]. Over the past ten years, several monodisperse magnetic nanoparticle preparations have been created for molecular and cellular targeting, cancer staging, immune cell tracking (monocyte/macrophage, T-cell), and angiogenesis imaging [114]. 18F-labeled fluorodeoxyglucose (FDG), a frequently used PET imaging probe, can only locate cancers by detecting body cells that have enhanced glucose absorption and metabolism, facilitating the identification of those tumors [115,116]. Cetyltrimethylammonium bromide is a cationic surfactant that is used in the synthesis of nanorods and is internalized into cells within hours [117]. When cationic surfactant cetyltrimethylammonium bromide is carefully removed from nanorods functionalized with folate, it causes the nanorods to deposit on the surface of the cell over the same period of time [118]. In either scenario, when exposed to radiation at the longitudinal plasmon resonance of the nanorods, these nanorods make the tumor cells very vulnerable to photothermal impairment, resulting in significant blebbing of the cell membrane [119]. Gold-coated nanoshells with a diameter of 120 nm have been used successfully by researchers to eradicate cancerous tumors in mice. By attaching peptides or antibodies to the surface of the nanoshells, it is possible to direct the nanoshells to bind to malignant cells [120]. Researchers have also created gold nanoparticles that are attached to an antibody that binds the EGFR cell surface receptor. The gold nanorods provide a bright, crisp picture solely for the tumor cells when treated with both normal cells and oral tumor cells and photographed using multiple distinct spectroscopic methods [121]. The optical signal from tumor cells was, in fact, two times as strong as that from healthy cells. Target-specific detection of biomarker molecules made only by cancer cells in conjunction with imaging probes and ligands that are capable of recognizing and interacting with target molecules is one of the most used molecular imaging techniques to increase the specificity of cancer diagnosis. Tumor-targeted magnetic, radioactive or optical probes have recently been developed, and their viability has been assessed in animal tumor models and in a few clinical investigations.

## 5. Nanotechnology-Mediated Cancer Therapy

Nanotechnology has made significant strides in the field of cancer therapy. New therapies have been created using the magnetothermal and photothermal effects of certain nanomaterials. For the imaging and diagnosis of tumor lesions, superparamagnetic materials can be used to obtain great sensitivity [122]. Additionally, the temperature may be raised to 40–45 °C in the presence of an external magnetic field to destroy tumor cells. For efficient biological treatment and medical cell operation, some complex tasks, like intracellular medicine administration, power production, and even detoxification of the blood vessels, can be accomplished by the application of nanomedical devices. Each NP in PDT has the capacity to transport numerous photosensitive molecules, making it possible to transfer a significant number of light-sensitive molecules to the tumor location. It can transport plenty of photosensitive chemicals into the tumor during PDT to destroy cancer cells. Various nanomaterials, particularly those used in semiconductors such as nanoscale titanium dioxide (TiO_2_) and zinc oxide (ZnO), have strong photocatalytic activity. Nanomaterials exhibit superior stability, low cost, excellent catalytic performance, and minimal cytotoxicity when compared to conventional photosensitizers. More crucially, it may target the tumor for destruction while sparing the healthy structure. PDT plays a significant part in the combination therapy for malignancies and broadens the photosensitizer’s application potential.

Apart from the conventional nanotechnological formulations, the research community of the biomedical realm have shown significant interest in myconanotechnology for drug delivery and development. Natural-based treatments have become a viable and safer substitute for synthetic medications for treating diseases like cancer. Due to the extensive ability of fungal endophyte to produce vital bioactive compounds, they stand out as one of the most remarkable microbial families with significant pharmacological importance [123,124,125,126]. The application of mass spectrometry in combination with bioinformatics tools has revealed the chemical and anti-breast cancer properties of the bioactive compounds produced from these fungal endophytes [127,128,129]. Moreover, the characterization of manufactured NPs and their biological use in diagnostics and treatments have relied extensively on microscopic methods [130]. The bioactive compounds derived from fungal endophytes may efficiently reduce metal ions, which attribute to their antioxidant potential [131,132,133]. Therefore, fungal endophyte derived bioactive compounds are employed to synthesize nanoparticles, thereby enabling efficient drug delivery and the potential for therapeutic applications in cancer. The mycogenic synthesis of silver nanoparticles, in particular, can be tailored as effective nanomedicine with promising potential in cancer treatment and medicine. Our recent study unveiled the green-based approach for bioengineering of silver nanoparticles involving the synthesis of silver nanoparticles mediated by bioactive compounds derived from *Penicillium oxalicum* (POAgNPs) which exhibited remarkable ability to enhance apoptosis in breast cancer cell lines [134]. Consequently, the bioengineering of silver nanoparticles mediated by fungal endophyte exhibit enhanced biocompatibility and may be implemented to target malignant cells.

### 5.1. Cancer Treatment Using Photodynamic Therapy-Based Nanotechnology

**Figure 3:**
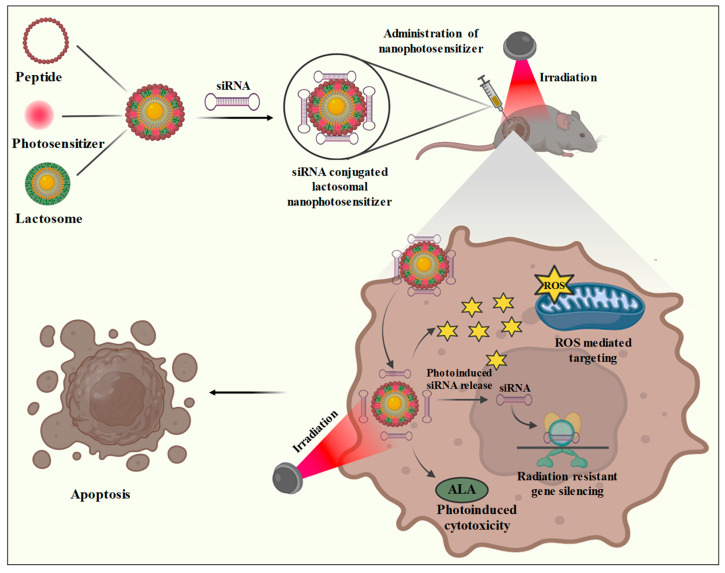
Lactosomal nanophotosensitizer mediated photodynamic treatment. Lactosomal nanophotosensitizer is designed by the incorporation of a polypeptide and photosensitizer conjugated with siRNAs. The lactosomal nanophotosensitizer upon administration is subjected to irradiation due to which the bound siRNAs are released and are responsible for radiation resistance gene silencing. The nanophotosensitizer is also responsible for the generation of ROS which promotes apoptosis. Additionally, phototreatment also induces the overexpression of ALA peptide resulting in ALA-mediated cell cytotoxicity.

Due to their typically high photocatalytic activity, semiconductor nanoparticles can be utilized as a photosensitizer in tumor photodynamic therapy (PDT). PDT is becoming a significant component of cancer treatment. When used with a photosensitizer, PDT can use a selective photodynamic response to eliminate local aberrant tissue, including tumors by elevating the production of ROS and ALA peptide (Figure 3). The main working principle is that the targeted molecules’ positioned photosensitizer is triggered to liberate reactive oxygen species (ROS), such as a single oxygen and oxygen-free radicals [135]. Numerous different biological substances can interact with ROS in tumor tissues and cells, causing cytotoxicity to arise and eventually causing the death of tumor cells and the elimination of tumor tissues.

Due to its distinct and adaptable features, Ag_2_WO_4_ has been employed in a variety of sectors and applications, including photodynamic treatment [136]. In recent research, an *in vivo* synthesis of leukocyte membrane coated GCMNPs (glycyrrhetinic acid encapsulated poly lactic-co-glycolic acid) was conducted to promote specific targeting, tumor-homing potency, and alleviate toxicity [137]. GCMNPs has the potential to subject the colorectal cancer and leukemia cells to ferroptosis by negatively regulating glutathione-dependent peroxidases 4 level, resulting in a high level of lipid peroxidation. Moreover, combinatorial effects of GCMNPs and ferumoxytol can increase Fe-mediated cytotoxicity. Similarly, phenethyl-conjugated chitosan oligosaccharide (ChitoPEITC) conjugates were designed for the decoration of chlorin E6 (Ce6)-integrated nanophotosensitizers for photodynamic treatment of HCT-116 colon cancer cell lines [138]. Recently, the synthesis of fluorinated CaCO_3_-based PFCE/hCe6@CaF-PEG nanophotosensitizer has gained significant interest in the realm of oncology. PFCE/hCe6@CaF-PEG mediated photodynamic therapy can inhibit the progression of primary CT26 tumors and unirradiated distant cancers in conjunction with local light emitting diode exposure [139]. On the other hand, for the treatment of cervical cancer, HAthCe6 nanophotosensitizers with CD44 receptor sensitivity can be administered in a receptor-specific way to target HeLa cells [140]. Nanophotosensitizers boosted ROS production, PDT effectiveness, and Ce6 uptake proportion in cultured HeLa cells as compared to free Ce6. The pre-treatment of free hyaluronic acid (HA) against HeLa cells lowered the Ce6 uptake ratio, ROS production, and PDT effectiveness of HAthCe6 nanophotosensitizers because HA preferentially interacts with the CD44 receptor of cancer cells. *In vivo* experiments in mouse models have demonstrated that bovine serum albumin (BSA) loaded photosensitizers (PS); PS@BSA can accelerate the Type I PDT machinery by producing significant amounts of superoxide radicals (O_2_^•−^) to enhance the PDT efficacy of hypoxic cancer [141].

PDT employs a variety of factors, including light, nano-photosensitizers, and oxygen, for cancer therapy. The conversion of molecular oxygen into reactive oxygen species takes place by the excitation of photon molecules which interact with the biomolecules of cancer cells and cause their destruction by inducing apoptosis. PDT and siRNA synergistically improve cancer treatment outcomes. Numerous studies have shown that siRNAs are highly effective at improving the responsiveness of cancer cells to PDT by targeting genes associated with autophagy [142]. The collective delivery of siRNA and an NP-based photosensitizer might be a crucial therapy method. It is feasible to efficiently and safely make T-cells immunogenic against cancer by silencing specific genes in immune suppressive pathways [143]. By specifically targeting the reduction in particular genes that lead to radiation resistance, siRNA treatment supports radiation therapy by improving the tumor induced response against radiation. A study demonstrated that the activation of AMPK by Metformin in lung cancer cells leads to the suppression of growth and an increase in sensitivity to radiotherapy [144]. Another well-known radiation resistance mechanism is an increase in DNA damage repair capabilities. Until now, there have not been any in-depth studies on siRNA co-delivery employing nanoparticle drug delivery systems, despite recent advances in nanomedicine exploring the application of nanoparticles for radio-sensitization.

A recent study showed the photodynamic treatment mediated by lactosomal nanoparticles improves cancer therapy by increasing the production of reactive oxygen species and silencing of the gene responsible for G2ABC transporter. A cell-penetrating peptide named L7EB1 was incorporated to lactosome particles to improve their absorption by cells. The G2ABC transporter (ABCG2) siRNA was coupled to an A3B-type lactosome to induce *in vitro* apoptosis of the NCI-H226 and PANC-1 cancer cell lines. The L7EB1 peptide does not carry siRNA into the cytosol, but it increases the efficacy of lactosome cellular absorption. A photosensitizer loaded L7EB1-lactosome was designed to evaluate the photoinduced cytosolic dispersion of siRNA, and the TPFPP (5,10,15,20-tetra-kis(pentafluorophenyl) porphyrin) photosensitizer exhibited remarkable photoinduction for cytoplasmic dispersion. In order to effectively transfer ABCG2 siRNA into the cytosol for gene silencing, the combined effects of improved cellular absorption by L7EB1 and TPFPP-based photoinduced endosomal escape were utilized [145].

The huge potential of photodynamic treatment (PDT) has sped up the development of photosensitizers (PS) that are incredibly effective in eliminating malignant carcinoma cells. Researchers have been working on developing new generation nano-photosensitizers with better photostability and higher single oxygen generation efficiency, as well as methods of improving the performance of existing photosensitizers, to mitigate the inherent limitations of the classical molecular photosensitizers. The first-generation PSs, which are made up of hematoporphyrin and its derivatives, have some structural restrictions because of their planar structure with abrupt organization that resulted from constant π-π stacking. On the other hand, second-generation PS exhibit limited tumor light penetration, extended skin photosensitivity in patients, and unsatisfactory tumor selectivity. Researchers have taken advantage of the benefits of non-toxic NPs in PDT in order to bypass the limitations of traditional PSs. Compared to biological PSs, noble metal plasmonic NPs have a multitude of advantages. It exhibits simplicity in synthesis, is characterized, and has surface functionalization. Gold and silver exhibit distinctive shape- and size-dependent optoelectronic characteristics. Furthermore, the increased extinction coefficients compared to conventional organic PSs were greatly improved by Ag and Au NPs. Plasmonic NPs stand out from other nanoplatforms including semiconductor quantum dots, magnetic, and polymeric NPs due to surface plasmon resonance.

### 5.2. Nanomaterial-Based Formulations in Reversal of Multidrug Resistance

**Figure 4:**
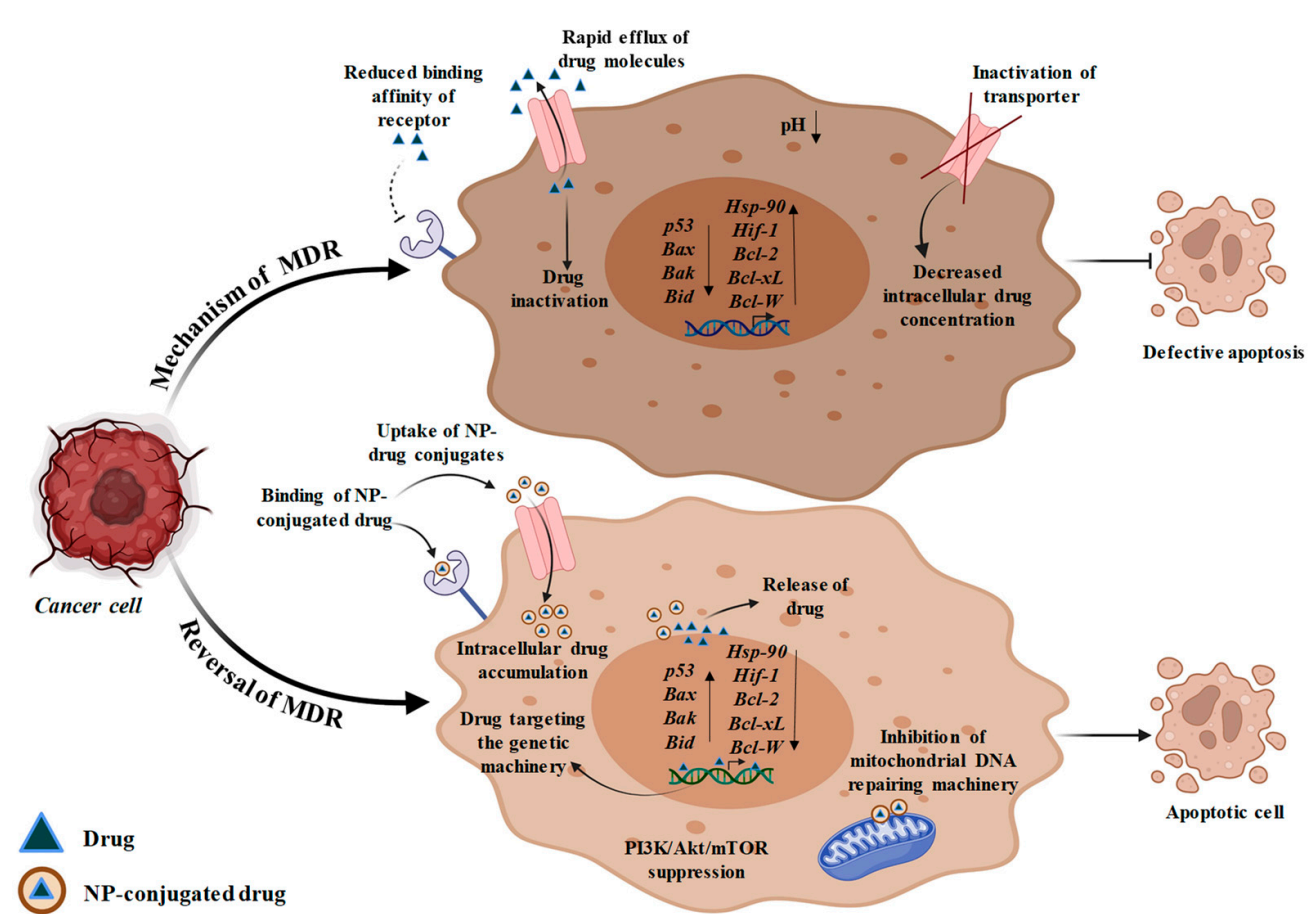
Mechanism of multidrug resistance and its reversal on treatment with NP-drug conjugates. MDR may occur as a result of reduced binding affinity of receptor with the drug. Hyper-activation of efflux transporters also leads to MDR causing rapid efflux of drug which results in reduced drug uptake and hence less intracellular drug accumulation takes place. MDR may also be caused due to defective apoptotic machinery or due to hypoxia which is responsible for overexpression of *Hif-1* gene and *Hsp90* gene. Other than this, the expression of tumor suppressor gene (*p53*) and pro-apoptotic genes (*Bax*, *Bak* and *Bid*) is reduced while the expression of anti-apoptotic genes (*Bcl-2*, *Bcl-xL*, *Bcl-W*) is increased. For MDR reversal, drug-loaded NPs are used that have higher binding affinity with receptors on a cancer cell which facilitates rapid uptake of drugs and its increased intracellular accumulation. The drug is afterwards released in the tumor cell due to low pH at the perinuclear surface which prevents the normal cells from system cytotoxicity, thereby resisting MDR. NP-loaded drug targets the genetic machinery and results in inhibition of *Hsp90* gene, which further causes suppression of *Hif-1* gene. Furthermore, the released drug also enhances the expression of *p53*, *Bax*, *Bak*, *Bid* and inhibits the action of *Bcl-2*, *Bcl-xL* and *Bcl-W* genes, which induce apoptotic machinery. The drug also targets P13K/Akt/mTOR pathway and mitochondrial DNA repairing machinery, resulting in mitochondrial DNA damage and release of Cyt-c, thereby inducing apoptosis.

One of the main challenges preventing the therapeutic effectiveness of biologic or chemotherapeutic drugs is drug resistance [146]. The overexpression of the ABC transporters (efflux transporter), defective apoptosis, acidic and hypoxic tumor microenvironment, and interstitial fluid pressure are the physiological and cellular factors that cause tumor drug resistance [147]. Nanotechnology has also served as a novel platform for naturally occurring P-gp inhibitors that have recently emerged as a potential adjuvant in chemotherapy for improving the pharmacokinetic attributes of chemotherapeutic drugs [148]. The blood-brain barrier, which prevents anti-cancer drugs from entering the brain from the outside, is one of the effective defense mechanisms induced by brain. However, the application of mesoporous silica nanoparticles has played a significant role in the treatment of brain cancer by crossing the blood brain barrier to deliver the drugs [149]. Furthermore, the role of selenium- and tellurium-based nanoparticles as an MDR reversal agents have also been reported [150]. The failure of numerous cancer therapies due to multidrug resistance results in disease progression and a poor prognosis. Drug resistance can be overcome in large part by using nanotechnology to deliver drugs for the treatment of cancer (Figure 4). Here, some of the tumor targeting strategies are described briefly in the subsequent sections.

#### 5.2.1. Efflux Transporters Targeting

Efflux transporters are members of the ABC transporter family that serve as a crucial contributor in the development of drug resistance. By pumping the drug out of the cell, efflux transporters lower its accumulation across cells, making therapy ineffective. One of the best studied efflux transporters is P-glycoprotein (P-gp), which is overexpressed in a number of drug-resistant cancers [151]. Since NPs primarily enter the cell through endocytosis instead of diffusion and liberate the drug at the periphery of the nucleus within the cell, away from efflux pumps, a number of studies have shown that some chemotherapeutic-loaded NPs can escape the subjection of anti-tumor drugs to efflux transporters [152]. By combining numerous therapeutic agents within a single drug carrier, NP-based combination treatment has been successful in overcoming this issue of pharmacokinetic variations between various medications, combating drug resistance, and enhancing the therapeutic efficacy of cancer therapy.

In addition to avoiding efflux transporters, another strategy for dealing with medication resistance brought on by these molecules would be to reduce their production and activity. This method can be carried out by creating NPs that include both chemotherapeutics and efflux pump inhibitors, or it can be accomplished by decreasing the amount of ATP delivered to the efflux pump. Since imatinib has been demonstrated to contribute to P-gp-mediated MDR in chronic myeloid leukemia (CML) K562 cell lines, administration of ketoconazole can suppress P-gp expression in CML cancer cell lines [153]. In a recent study, the combination of DOX and miRNA-495 in a tumor cell membrane-coated silica NP successfully overcame MDR in lung cancer therapy [154]. The study’s findings showed that miRNA-495 effectively downregulated P-gp expression in multidrug-resistant oncogenic cells [155].

#### 5.2.2. Apoptosis Targeting

Cancer drug resistance is a result of defective apoptotic machinery that allows cancer cells to avoid apoptosis and improve survival. The *Bcl-2* and nuclear factor kappa B (*NF-KB*) pathways are frequently dysregulated, which activates the defective apoptotic machinery. *Bcl-2* is a thoroughly researched anti-apoptotic protein that is also a major contributor to drug resistance [156]. These factors together imply that *Bcl-2* may be a good target for reversing medication resistance. The option to overcome treatment resistance in cancer has emerged as the co-delivery of chemotherapeutic drugs and *Bcl-2*-targeted siRNA via NPs [157]. Pro-apoptotic chemicals can also be activated to fight apoptotic pathway-dependent drug resistance in addition to inhibiting anti-apoptotic proteins. For instance, combining a natural compound; rubusoside with the C6-ceramide improves the therapeutic effectiveness of several tumor models that are resistant to different types of drugs [158]. On the other hand, according to a recent study, it was found that ceramide can control alternative splicing of pre-mRNA to restore the production of the vital tumor suppressor *p53* protein in its wild-type form [159]. Restoring the function of *p53* or other tumor suppressors is considered as a possible strategy for combating cancer drug resistance since *p53* plays a substantial role in apoptosis. As a result, more research has been conducted on *p53* gene therapy using a nanoparticle-based delivery method. In lung and breast cancer cells, PLGA and cationic solid lipid NPs have been shown to differentially induce the *p53* gene [160]. These findings demonstrate potent apoptosis induction and tumor growth suppression.

#### 5.2.3. Hypoxia Targeting

Hypoxia is also one of the major factors that contribute to MDR. Cancer cells are often in a state of hypoxia so as to meet the oxygen demands of hyperproliferating cancer cells. Drug resistance can be influenced by hypoxic conditions in various ways. Cancer cells undergoing slow division in an area of limited oxygen supply often resist cytotoxic chemotherapy drugs, including alkylating agents and antibiotics [161]. Hypoxia-inducible factor 1 (*HIF-1*) is crucial to the process and has been found to be overexpressed in a variety of human malignancies. In order to combat drug resistance, a strategy must be made to execute the targeting of *HIF-1*. The silencing of the *HIF-1* gene by the application of nanomaterial-based *HIF-1* siRNA is one of the methods to cope with the hypoxic condition [162]. The expression of the *HIF-1* gene can be downregulated by indirect inhibition of the PI3K/Akt/mTOR signaling pathway, which results in improved efficacy of cancer therapy against MDR [163]. In this approach, NPs such as PLGA-PEG and liposomes can provide superior platforms for combination treatment. In addition, *HIF-1* transcriptional activity depends on heat shock protein 90 (HSP90), and HSP90 suppression can reduce HIF-1 expression [164]. It has been demonstrated that the HSP90 inhibitor in 17AAG-loaded NPs significantly enhances bladder cancer therapy [165].

Moreover, in a recent study the synthesis of hollow nanoparticles consisting of conjugated oligomers proved to be effective phototheranostic agents for targeted PDT (photodynamic therapy) against hypoxia [166]. A conjugated oligomer serves as the photosensitizer while an amphiphilic polymer serves as the matrix in the simple nanoprecipitation process used to create the sialic acid (SA)-imprinted hollow nanoparticles. The π–π stacking interactions control the self-assembly and results into the development of hollow nanostructures. Apart from this, the application of hypoxia-responsive element (HRE) driven therapeutic cargo expression as a method to induce the expression of genes specific to cancer has gained much attention. This method allowed for the hypoxia-specific production of two therapeutically important cargo components, namely the CRISPR-Cas9 nuclease and the HSV-tk suicide gene [167]. An HIF inhibitor; PX478 when conjugated with silk fibroin nanoparticles were reported to cope with MDR by increasing DOX efficacy to MCF-7/ADR cells [168]. Furthermore, plasmid-integrated lipid nanoparticles were used to deliver therapeutic cargo to tumor cells with high efficiency. This allowed for the targeted killing of tumor cells in hypoxic environments while maintaining tight regulation and no appreciable shifts in cell viability in normoxia.

### 5.3. Role of Nanotechnology in Cancer Immunotherapy

In addition to chemotherapy, nanotechnology also makes a significant contribution to enhancing immunotherapy for cancer treatment. The use of nanovaccines and artificial APCs is part of NPs-based immunotherapy. A wide range of adjuvants and tumor-associated antigens are delivered to the APCs, (dendritic cells) using nanovaccine [169]. This leads to improved DC maturation and antigen presentation, which result in the stimulation of anti-cancer activity in cytotoxic T-cells. Among many kinds of NPs, it has been demonstrated that inorganic NPs like acetylated dextran (AcDEX) and mesoporous silica can act as adjuvants in immunotherapy, stimulating the immune response [154]. Moreover, nanotechnology has also contributed in the chemo-immunotherapy of breast cancer cells [170]. Furthermore, the combination of immunotherapy and chemotherapy is considered a better and more efficient method for cancer treatment [171]. A recent study found that co-loading the cytokine GM-CSF and the chemotherapeutic agent Nutlin-3a in spermine-based AcDEX NPs enhanced the cytotoxic CD8(+) T-cell proliferation and accelerated the immunogenic response, leading to tumor cell destruction while avoiding toxicity in immune cells [172]. In addition to this, co-delivery of monoclonal antibodies and chemotherapeutics into porous silicon nanoparticles is another alternative technique to combination chemo-immunotherapy that has been successful in promoting antibody-dependent cell cytotoxicity (ADCC), complement activation, and an immune response against cancer cells [173].

### 5.4. Nanotechnology Mediated CRISPR/Cas9 Delivery for Cancer Therapy

Gene delivery by the aid of viral vectors have been somewhat successful in delivering proteins and nucleic acids, but they still need to be made more effective in terms of efficiency, tissue targeting, and toxicity. Nano-vehicles can be utilized as an appropriate mechanism to overcome obstacles and can also be employed as novel methods for genome editing for cancer therapy due to their safety and efficacy in clinical settings. The development of extremely effective therapy regimens that work in clinical situations can be assisted by such predicted substitution. CRISPR/Cas9 completely suppresses the Bcl-2-mediated anti-oxidative stress system as compared to Bcl-2 inhibitors and RNAi technologies. Target tissues are often accessed by the CRISPR/Cas9 system via safe and efficient means. The incorporation and delivery challenges of vectors have significantly increased because of CRISPR/Cas9 plasmid. In order to deploy the CRISPR/Cas9 system, it is therefore challenging to build a safe and highly effective vector. A tumor-specific induced nano-domino-CRISPR (TAN) co-administered with the chlorins e6 (Ce6) photosensitizer and the CRISPR/Cas9 plasmid encoding the Bcl-2 gene is purposefully engineered and constructed to disrupt the anti-oxidative stress and trigger apoptosis to deliver a customized therapy [174]. In particular, nanoscale delivery systems can efficiently transport CRISPR/Cas9 while allowing gene therapy to be combined with other treatment modalities, promoting the development of nanotechnology-based CRISPR/Cas9 system-mediated cancer therapy. For instance, a CRISPR-mediated CD47 blockage and IL-12 generation in tumor cells are combined utilizing a particular response-stimulated delivery of nanosystems in a gene editing immunotherapeutic approach [175]. Recent research also revealed the use of CRISPR/Cas9 to investigate potent distinct gene dependencies in response to various poly (ADP-ribose) polymerase inhibitor (PARPi) and platinum-based chemotherapy [176].

### 5.5. RNA-Mediated Cancer Nanotherapy

RNA nanotechnology has emerged as a multidisciplinary and constantly emerging platform in the field of cancer biology because of its versatile surface attributes that make it suitable to be employed for the designing of a diverse range of nanomaterials. In the field of RNA nanotechnology, mRNA, siRNA, miRNA, sgRNA are some of the therapeutic RNAs that have remarkable potential to suppress tumor by upregulating the tumor suppressor genes and downregulating the expression of oncogenes. Among the non-coding RNAs, miRNA control the expression of their target RNAs by causing RNA cleavage, destruction, or preventing RNA translation [177]. Long non-coding RNAs (lncRNAs) have been shown to be essential regulators of renal cell carcinoma (RCC) progression. In order to deliver lncRNAs, RCC cells were transfected with the plasmid-encoding tumor suppressor lncRNA-SLERCC (SLERCC). Both *in vivo* and *in vitro* tests were performed on the Plasmid-SLERCC@PDA@MUC12 nanoparticles (PSPM-NPs), which included cellular uptake, CCK-8 assay, tumor growth suppression, histological evaluation, and safety assessments. These results demonstrated that SLERCC is an intriguing target for cancer therapy and that transmembrane metastasis markers-targeting plasmid-encapsulated nanomaterials may provide a new therapeutic approach for RCC therapy [178]. In contrast to this, accumulating evidence also validates that a TLNC1; a lncRNA is responsible for progression and metastasis of hepato-carcinoma cells by silencing p53 mediated apoptosis [179].

## 6. Application of Nanotechnology in Cancer Stem Cells (CSCs)

Recent advancements in preclinical and clinical cancer research have led to the development of new diagnostic and therapeutic alternatives, which would significantly improve cancer therapy and prevention. The property of cancer cells to undergo metastasis and spread from localized tissue to other areas has restricted the efficacy of conventional therapeutic methods. Some heterogeneous groups of cells arise from mutations of pre-existing normal stem cells, and these are referred to as ‘cancer stem cells’ (CSCs). The undifferentiated normal stem cells have self-renewal properties and they differentiate to form any type of cell. However, CSCs differentiate and proliferate to form malignant cells that can undergo rapid expansion and spread to other tissues, thereby taking the form of metastatic cancer. CSCs cannot be easily destroyed by the use of conventional cancer treatment methods and are responsible for cancer relapses at later stages. Therefore, the detection and eradication of CSCs have been a major challenge in the field of cancer research. The utilization of nanoscale materials for CSC-directed anti-cancer treatments is therefore of great interest.

Recently, a nanosensor-mediated detection of CSCs derived extracellular vesicles was developed to overcome the obstacles related to liquid biopsy [180]. Some biomarkers can be used to identify CSCs, which will enhance treatment methodologies and therapeutic results. A diagnostic CSCs biomarker in salivary gland tumors was recently assessed using gold nanoparticles that were designed and integrated with CD24 primer to create a CD24-AuNP [181]. Similarly, the CEACAM 6 (CD66c) antibody, a native biomarker of breast CSCs, was also employed for the synthesis of a liposome-based nanocomposite. Depending on the amount of CD66c expression, breast CSCs on treatment with CD66c Ab-conjugated rhodamine-labeled liposomes (CDRHOL) indicated selective and enhanced cellular absorption [182]. Researchers have demonstrated that nanoparticle-based curcumin delivery has enhanced its bioavailability and resulted in the eradication of CD133 + CSCs, thereby inhibiting the growth of brain tumors [183]. The overexpression of CD133 in CSCs of osteosarcoma has been targeted by the application of CD133 aptamers conjugated with salinomycin loaded PLGA nanoformulations [184]. Furthermore, targeting specific signaling pathways such as Hedgehog, Notch, and Wnt/-catenin has been shown to be effective in destroying CSCs [185].

## 7. Future Perspectives and Concluding Remarks

The present review focuses on the latest developments in the field of nanotechnology-mediated diagnosis and treatment of cancer, as nanotechnology has the potential to facilitate the early detection, prediction, and prevention of cancer during its premalignant phases. In the battle against devastation caused by cancer, the field of nanotechnology enables delivering multimodality treatment which is not achievable with existing conventional procedures. Nanotechnological applications, for instance, tissue regeneration, immunoassay, removal of toxins, drug administration, hyperthermia, and cell sorting have all been successfully carried out due to the surface chemistry of nanoparticles. The clinical effectiveness of radio-labeled monoclonal antibodies in the cancer diagnosis and treatment portends well for the use of nanocarrier systems in medical oncology in the future. In animal models, a number of radio-labeled multifunctional nanocarriers have shown better efficacy in the detection and management of cancer. However, more preclinical, medical, and high degree toxicity research will be needed for the application of this technique for the treatment of cancer patients. The majority of the scientific research in this field has shed light on enhancing biocompatibility of materials; however, very few scientific studies and initiatives have been taken to enhance the standard and shape, size and surface characteristics of magnetic particles to obtain a procedure for their quality control. In the near future, cell healing technologies and neuro-electronic interfaces may revolutionize the medical field when used for brain tumors, but for the time being, nanomedicine is quickly growing to become one of the largest global enterprises. The most significant advancements are occurring in drug delivery, which entails creating nanoscale molecules or particles to increase bioavailability. The goal of drug delivery is to enhance the bioavailability of medications by ensuring their targeted release at a specified site and over an extended period of time.

In the near future, advancements in the realm of nanomedicine will offer a valuable array of research tools and therapeutically beneficial devices. It is crucial to have a better in-depth understanding of the basic concepts applied in designing and using nanoparticles for diagnosis, treatment, or theranostics (combination of imaging diagnosis and therapeutics) in various medical scenarios, especially in light of the promising advancements in the expansion of imaging and nanotherapeutic approaches to cancer detection and therapy. The establishment of characterization tools and the regular updates regarding the latest clinical data are prerequisites for the deployment of nanomaterials. The impact of nanomaterials on patients as a result of the clinical trial has not been sufficiently explored to date. Moreover, despite all of the limitations, the general consensus is that, beyond the realm of oncology, nanomedicine has the potential to revolutionize various other biomedical domains. Scientists will persistently strive to develop novel and efficient nanosystems to diagnose and treat cancer, driven by the quick and promising advancements in nanotechnology.

## Figures and Tables

**Table 1:** Different types of nanoformulations and their biological applications, along with advantages and disadvantages.

S. No	Nanomaterials-Based Drugs	Biological Application	Advantages	Disadvantages	References
1.	Linalool encapsulated solid lipid NPs	Anti-cancer efficacy against HepG2 and A549 cancer cell lines	Enhanced cellular uptake, better tumor inhibitory effects	Uncertain gelation tendency, low rate of incorporation due to crystalline nature	[13]
2.	PCL-Tween80 polymeric NP	Improved anti-tumor efficacy	Better internalization, enhanced cellular uptake as compared to free form	Lack of *in vivo* investigations	[14]
3.	C225 antibody conjugated AuNP	Noninvasive radiofrequency-based hepatocellular cancer treatment	Enhanced thermal cytotoxicity, better intracellular accumulation, good stability	Limited radiofrequency absorption	[15]
4.	CD44 antibody targeted liposomal nanoparticle	Immunoliposomal imaging and therapy against hepatocellular carcinoma	Improved drug delivery and enhanced imaging	Low specificity and efficacy	[16]
5.	Mit-loaded liposome (Mit-GML)	Image guided targeted cancer therapy against MCF-7 breast cancer cell lines	Increased cellular uptake, better intracellular accumulation	Reduced cytotoxicity against cancer cells than free form	[17]
6.	HPTOC-DOX polymeric micelle	Enhanced anti-cancer efficacy	Synergistic effect of TTP and DOX, redox-sensitive drug release, site-specific targeting	Low cellular uptake, short shelf life	[18]
7.	PP-SS-DTX/DTX polymeric micelles	Anti-cancer activity against MCF-7 and B16F10 cancer cell lines	Better cytotoxicity, high stability, stimuli-sensitive drug release	Delayed drug release	[19]
8.	Curcumin loaded PMMA-PEG/ZnO	Anti-angiogenic and anti-proliferative activity against gastric cancer	Improved pharmacokinetic properties	Limited solubility and biological stability	[20]
9.	Anti CD147 immunoliposomal DOX	Target the CD147 overexpressing hepatocellular carcinoma	Increased intracellular accumulation, better binding and internalization	High manufacturing cost, sophisticated synthesis, lack of deep penetration	[21]
10.	DOPE/CHEMS-based DTX loaded immunoliposomes	Targeted delivery of DTX for the treatment of prostate cancer	Better DTX encapsulation, controlled drug release, pH-resistant formulation	No relevant cytotoxicity	[21]
11.	YIGSR-CMCht/PAMAM dendrimer nanoparticles	Targeted therapy against colorectal cancer	Less side effects, antiproliferative activity against cancer cells	Lack of promising investigation to validate the specific affinity	[22]
12.	ICG-Loaded PEGylated BSA-Silver Nanoparticles	Effective photothermal cancer therapy	Good photostability, safe to use, non-toxic, non-immunogenic, biocompatible	Ineffective at lower concentration	[23]
13.	TT3-*o*CB NP@EXOs exosomal NP	Image guided photothermal tumor therapy	Biocompatible, chemically stable, enhanced intercellular communication	Toxic degradation of polymeric NP results in the entry of toxins in CNS	[24]
14.	*Aspergillus austroafricanus* CGJ-B3 AgNP	Cytotoxic activity against MCF-7, A431, and HepG2 cancer cell lines	Antioxidant activity against ROS and RNS for treatment of neurodegenerative diseases	Lack of toxicity related *in vivo* and *in vitro* investigations	[25]
15.	Amygdalin loaded AgNP encapsulated polygonal chitosan microcapsules	Targeted delivery of amygdalin for breast cancer therapy	Biocompatible, biodegradable, non-toxic, can be easily modified, hydrophilic, permeable	Lower cytotoxicity as compared to free amygdalin loaded AgNP	[26]

## Data Availability

Data sharing option is not applicable, as no new data were generated or analyzed in this review.
